# The Metropolitan Hospital Sunday Fund

**Published:** 1887-06-18

**Authors:** Sydney Waterlow

**Affiliations:** Vice-President of the Council


### THE METROPOLITAN HOSPITAL SUNDAY FUND.

By Sir Sydney Waterlow, Bt., Vice-President of the Council.

In speaking, on Wednesday week, on behalf of the Hospital Sunday Fund, at the Hampstead district meeting, Sir Sydney Waterlow said: It affords me great pleasure to say a few words in support of an object for which I have been working for fourteen years. I originated this Fund in 1873 at the suggestion of *The Lancet*, and I have had the privilege of having been Chairman of the Committee ever since the Fund was first collected. There was a great difficulty in the beginning in distributing—so as to give satisfaction—the money collected, among 150 hospitals, dispensaries, and convalescent homes, a difficulty almost as great as that of collecting the money itself, but now the Fund is distributed on a principle which commends itself to all. We commenced last year a series of meetings which were a great success. They were started from a feeling that the public generally did not grasp the duties and the responsibilities which fell upon them in supporting the hospitals and dispensaries of the country in which they lived. In America the maintenance of the hospitals and dispensaries is cast as a tax upon the population. It is not so in this country, and I hope it may never be, but that the voluntary principle will always be maintained. We have in the metropolis three or four large hospitals, mainly supported by endowments, but the others are dependent very largely upon voluntary contributions from the public. As I have said, I do not think the public thoroughly comprehend the duty devolving upon them. I want, now, to make everyone feel that that which they have to lean upon in the time of their trouble they should not forget in time of health. Let us look for a moment at the number of institutions in the north and north-west district of London. Here there are eleven hospitals and dispensaries, whose total expenditure last year was £60,346, while the total income was only £52,381, so that, even in this section of London, they had to fall back on their savings to the extent of £8,000. If that went on they would soon have to shut their doors. I am quite sure the public would not allow these institutions to fall into that condition if they understood the necessity for supporting them—they do understand the necessity for having them there when they want them. The cabmen are advertising the Hospital Saturday and Sunday Funds, and it is quite right. Very frequently the cabs, from accidents, send a great many people to our hospitals. There used to be a time when it was thought you could not get a patient admitted to a hospital without a governor's ticket. Now, however, in the great majority, the doors are never closed against an urgent case.

The resolution which I am seconding tells you that you are to give on Hospital Sunday, owing to the nominal cost of collecting. I am very glad to be able to tell you that the average cost is only three per cent. on the amount collected. Now, there is not a single institution which has to do its own collecting, which can do it at anything like that cost. Therefore, I want you to feel that, whether you are giving a penny, or a pound, or a five-pound note, all of it, practically speaking, goes to the object intended to be benefited. Our chairman (Lord William Compton) has told you that we ought to collect a great deal more. Through the kindness of more than 2,000 ministers of all denominations collections are made, and I think you will feel with me that the £40,000 collected last year—including £7,000 or £8,000 given in specific cheques—is not what we ought to collect in 2,000 churches in a metropolis of five million inhabitants. There is another reason which stimulates me to support the Hospital Sunday Fund in London. Whatever differences we may have on other subjects, on Hospital Sunday we are all of one mind, and go to church to pray to our Heavenly Father to do what is wisest and best for this object. All our religious differences are sunk. Now this is a glorious thing to think of on one Sunday in the year.

Let me for one moment call your attention to the very large sum which has to be collected in order to support the hospitals, dispensaries, and convalescent homes. In 1876 the income of 73 London hospitals was £192,736, but their expenditure was a great deal more than that. When the Hospital Sunday movement was commenced, hospitals, dispensaries, and convalescent homes were much fewer in number. They have grown just 50 per cent. since 1873, and the convalescent homes have increased in very much larger proportion, for there has at last come over the public a feeling that the work of the hospital is not completed when the acute symptoms of the patients are cured, or when the poor man has just had some serious surgical operation performed. Hence the number of convalescent homes has increased. Patients are sent to these, staying from two to four weeks, obtaining rest, food, and air, and going back to their families thoroughly restored in health. The value of convalescent homes is thoroughly understood now. These institutions have, however, thrown a large additional claim upon the Fund annually collected on Hospital Sunday, a claim which I rejoice to see put forward, because this means of ameliorating the sufferings of the poor has been largely increased. I trust the time is not far distant when every hospital will have in connection with it a convalescent home to complete the cure of patients under its care.

